# Identification of Up-Regulated ANXA3 Resulting in Fracture Non-Union in Patients With T2DM

**DOI:** 10.3389/fendo.2022.890941

**Published:** 2022-06-24

**Authors:** Changjiang Liu, Yuhang Liu, Yifeng Yu, Yong Zhao, Dong Zhang, Aixi Yu

**Affiliations:** Department of Orthopedics, Zhongnan Hospital of Wuhan University, Wuhan, China

**Keywords:** weighted gene co-expression network analysis, bioinformatics, GWAS analysis, single-cell RNA-sequencing analysis, T2DM, fracture non-union

## Abstract

Diabetes mellitus is a metabolic disorder that increases fracture risk and interferes with bone formation and impairs fracture healing. Genomic studies on diabetes and fracture healing are lacking. We used a weighted co-expression network analysis (WGCNA) method to identify susceptibility modules and hub genes associated with T2DM and fracture healing. First, we downloaded the GSE95849, GSE93213, GSE93215, and GSE142786 data from the Gene Expression Omnibus (GEO) website, analyzed differential expression genes and constructed a WGCNA network. Second, we screened out 30 hub genes, which were found to be enriched in neutrophil activation, translational initiation, RAGE receptor binding, propanoate metabolism, and other pathways through Gene Ontology (GO), Kyoto Encyclopedia of Genes and Genomes (KEGG), and gene set enrichment analysis (GSEA) analyses. Third, we searched for genes related to bone metabolism and fracture healing in the published genome-wide single nucleotide polymorphism (SNP) data, built a protein-protein interaction (PPI) network with hub genes, and found that they were associated with metabolic process, blood vessel development, and extracellular matrix organization. *ANXA3* was identified as the biomarker based on gene expression and correlation analysis. And the AUC value of it was 0.947. Fourth, we explored that *ANXA3* was associated with neutrophils in fracture healing process by single-cell RNA sequencing analysis. Finally, we collected clinical patient samples and verified the expression of *ANXA3* by qRT-PCR in patents with T2DM and fracture non-union. In conclusion, this is the first genomics study on the effect of T2DM on fracture healing. Our study identified some characteristic modules and hub genes in the etiology of T2DM-associated fracture non-union, which may help to further investigate the molecular mechanisms. Up-regulated *ANXA3* potentially contributed to fracture non-union in T2DM by mediating neutrophils. It can be a prognostic biomarker and potential therapeutic target.

## Introduction

Diabetes mellitus (DM) is a long-term metabolic disease characterized by high blood glucose levels. It is estimated that approximately 463 million people worldwide are suffering from diabetes in 2019 and this number will exceed 700 million by 2045 (1, 2). In the UK, £19,000 is spent on diabetes treatment per minute and this leads to negative effects on the economy and community health (3, 4). In addition, more than half of people with diabetes are initially unaware of having diabetes, making them more likely to develop complications (5, 6). Among these complications, expecting the well-known macrovascular and microvascular risks, DM is also recognized to negatively affect the bone material quality and fracture healing (7, 8). Type 2 diabetes mellitus (T2DM) is associated with an increased risk of all-cause mortality, including fractures (9, 10). However, clinical features and poor blood glucose control are not good predictors of diabetic complications, while numerous studies have shown that diabetes and complications do have clear genetic indicators (11). Therefore, genetic studies associated with diabetes may be of great significance.

Fracture healing process is a complex biological process. Physiological fracture healing includes inflammation, regenerative healing, and bone reconstruction processes (12, 13). Most fractures remodel to normal within six to eight weeks. However, if the fracture healing environment is incomplete, it may cause fracture delayed healing or even non-union (14). Fracture non-union is defined as a fracture that does not heal within nine months, and there is no sign of healing for three consecutive months (15). The prevalence of non-union is 5%-10% in fracture patients (16, 17). Many factors may cause fracture healing disorders, among which diabetes not only increases the risk of fractures, but also delays fracture healing time (approximately 87%) by regulating bone metabolism and the development of microvascular diseases (18). Although there were some articles on diabetes and poor fracture healing (19, 20), studies based on high-throughput gene sequencing profiles have not been reported. To fill this gap, we designed and implemented this research.

Weighted gene co-expression network analysis (WGCNA) was proposed to analyze the relationship between modular genes rather than an individual gene and clinical feature subtypes (21). It plays an important role in many biological research, such as in tumors (22, 23), immune diseases (24), chronic obstructive pulmonary disease (COPD) (25). It is a good tool for the analysis of genes associated with clinical characteristics and to screen biomarkers. Single-cell RNA sequencing (scRNA) analysis is an innovative research method that studies gene expression differences between groups by clustering single cells in samples, and have been used in tumors and non-tumor diseases (26). Here, we applied the WGCNA method to analyze trait-related genes between T2DM and fracture non-union, performed biological function correlation analysis, identified the biomarker gene *ANXA3*, and utilized scRNA analysis to explore its role in fracture healing, and finally validated the expression level of *ANXA3* with samples from patents with T2DM and fracture non-union in the local hospital. To the best of our knowledge, we performed the first genomics study on this research topic.

## Materials and Methods

### Data Resource and Criteria

**Figure 1:**
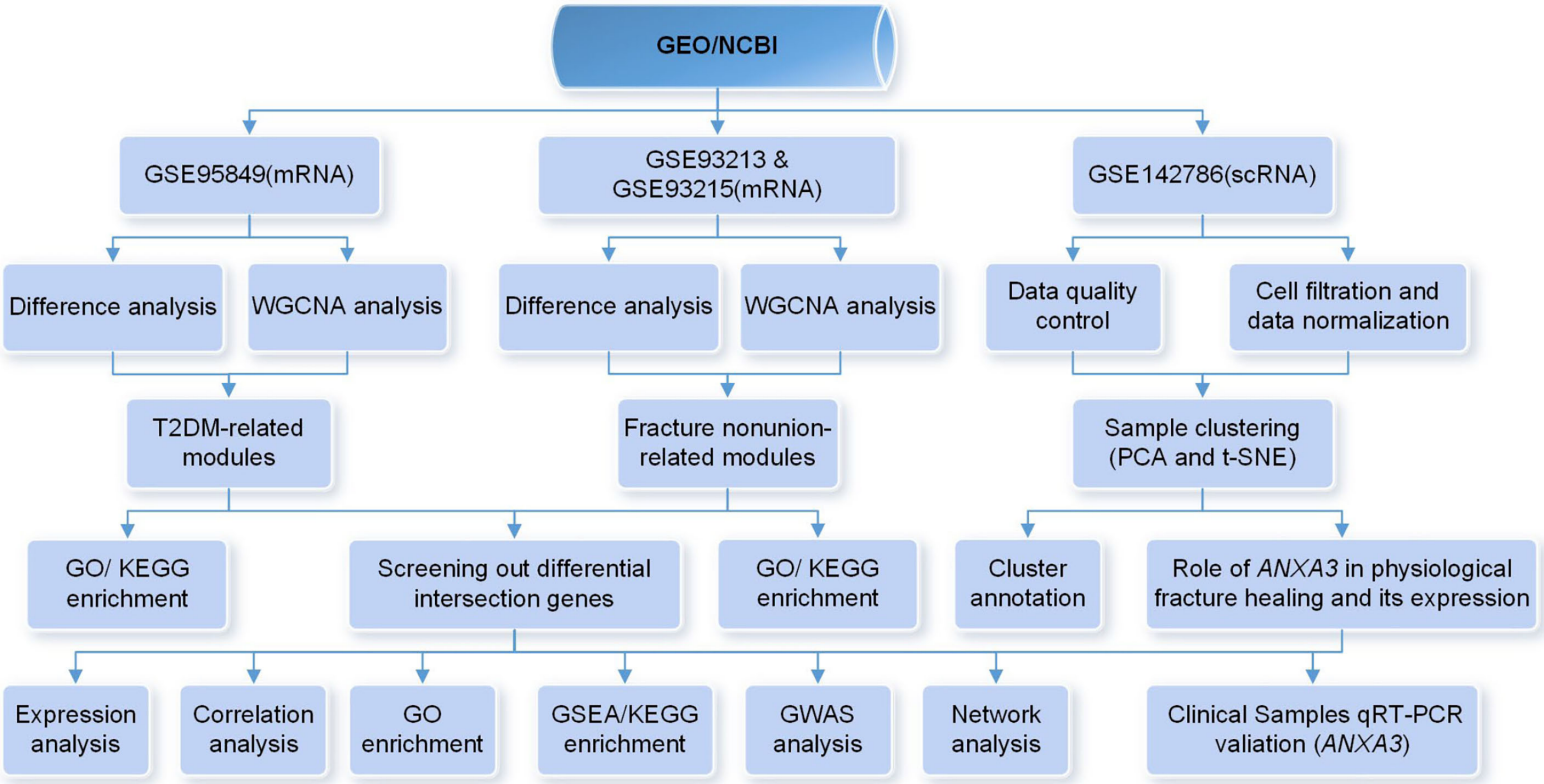
Flow chart of the whole procedures in this study.

To explore the potential relationship between T2DM and fracture healing, we systematically searched for relevant high-throughput functional genomic expression matrixes in the Gene Expression Omnibus (GEO) database (https://www.ncbi.nlm.nih.gov/gds/), a comprehensive database containing data on various diseases. During the mRNA expression data selection process, we developed several inclusion criteria as follows: (1) The species studied was *Homo sapiens*; (2) The sample types in the related data matrices which will be conducted the same analyses were consistent; (3) All data were publicly available and usable. Finally, three mRNA matrices and one scRNA matrix were selected for the next step of the analysis. The overall procedure of this study was shown in **Figure 1**.

### Differential Expression Analysis in the GEO Matrix

We used the “limma” R package for data quality control, processing, and statistical analysis. A multi-array average (RMA) method was performed to normalize gene expression profiles. The patients were divided into the T2DM group or normal group, fracture healing group or non-union group according to their clinical features. Differential expression genes (DEGs) were identified with a false discovery rate (FDR) < 0.05 and a |log2 fold change| ≥ 1.

### Identification of T2DM-Related and Nonunion-Related Genes by Weighted Gene Co-Expression Network Analysis

We constructed a gene co-expression network using the WGCNA package, which is suitable for identifying genes for specific phenotypic modules (T2DM-related and nonunion-related features) (27). Firstly, we calculated the Pearson correlation coefficient (PCC) for all paired genes and constructed an adjacency matrix to strengthen strong correlations between genes and penalize weak relationships. Second, we transformed the adjacency matrix into a topological overlap matrix (TOM) and performed average linkage hierarchical clustering to cluster similar gene classes into modules. Third, we calculated gene significance (GS) and module membership (MM) to associate modules with clinical features. Finally, we visualized the network of co-expression modules. Module-associated genes were used for the next step of the analysis.

### Screening of Hub-Related Genes and the Correlation Analysis

Based on co-expression network analysis, the “VennDiagram” R package was used to intersect highly module-related genes with DEGs to further obtain differentially expressed trait-related module genes associated with T2DM and fracture non-union. The expression profiles of hub genes in the non-union and T2DM GEO matrices were extracted. We then used the “heatmap” R package to map the genes expression heatmap and the “corrplot” R package to calculate the PCC between gene transcript levels in this GEO matrix. These genes were also searched in the STRING online database (https://cn.string-db.org/, version 11.5) and protein–protein interaction (PPI) network diagram was drawn to explore the correlation of these genes online.

### Gene Ontology Functional Annotation and Gene Set Enrichment Analysis

To further understand the biological functions of module-related DEGs and hub genes in T2DM and non-union, we performed GO functional enrichment analysis and Kyoto Encyclopedia of Genes and Genomes (KEGG) analysis using the “clusterProfiler” R package. The thresholds were P < 0.05 and FDR < 0.05. We also performed GSEA analysis between the fracture healing and non-union groups to validate these key genes. Gene set permutations were performed 1000 times for each analysis. The thresholds for data filtering were p-value < 5% and q-value < 25%.

### Genome-Wide Association Study Related Analysis

GWAS analysis was used to identify disease susceptibility genes (28). We searched the published susceptibility GWAS genes associated with fracture healing, explored the correlation with hub genes in the STRING online database, and visualized the result by using Cytoscape software (version 3.8.2). Biological functional enrichment between key genes and GWAS genes was investigated using the Metascape online tool (https://metascape.org/gp/index.html). In addition, we extracted the expression data of hub gene in matrix profiles of T2DM and bone nonunion and plotted a violin plot using the “ggpubr” R package. The “pROC” R package was used to plot Receiver Operating Characteristic (ROC) curve for hub gene.

### Single-Cell RNA Sequencing Analysis in Fracture Healing Process

To explore the potential role of biomarker genes in fracture healing, we performed single-cell sequencing analysis in GEO matrix. First, we calculated the percentage of mitochondrial genes in cells by using the “PercentageFeature” and filtered cells by mitochondrial gene percentage < 5% and gene expression counts > 50. Secondly, we analyzed the correlation between gene features and sequencing depth to judge data reliability. Then 500 highly variable genes were selected for analysis, and the value of principal components (PCs) was set to 20 to obtain cell clusters. Which will be displayed by “tSNE” R package. Thirdly, “singleR” R package were applied for cell annotation. Finally, we displayed the expression of biomarker genes in clusters, and utilized “VlnPlot” package to show gene expression levels in two groups.

### Validation of Hub Genes Expression and Quantitative Real-Time PCR Analysis

To verify the expression levels of biomarker genes in patients, we collected the blood samples from patients in Zhongnan Hospital (Hubei, China). They were divided into four groups: fracture healing without T2DM(n=3), fracture healing with T2DM(n=3), non-union without T2DM(n=3), non-union with T2DM(n=3). We used red blood cell lysis solution (Solarbio, China) to lyse red blood cells. Cell precipitates were collected by centrifugation at 450 rpm for 10 min, which were further lysed with TRIzol Reagent (TIANGEN, China). RNA was extracted and purified using chloroform, isopropanol, and ethanol solutions. We used Nanodrop 2000 to detect RNA concentration and adjusted the final RNA concentration to 1000 ng/µL. Next, the RevertAid First Strand cDNA Synthesis Kit (Thermo, German) was utilized to reverse transcribe RNA into cDNA. Finally, we performed qRT-PCR analysis of cDNA by FastStart Universal SYBR Green Master (Roche, Switzerland). *ANXA3* gene primers: 5’-ACCGCGCTTTGGATTAGTGT-3’(forward), 5’-CAGCATCCACTGATGGGCTA-3’(reverse). GAPDH gene primers: 5’-GAGAAGGCTGGGGCTCATTT-3’ (forward), 5’-TAAGCAGTTGGTGGTGCAGG-3’ (reverse).

### Statistical Analysis

All data were analyzed with R 3.6.3 software. Graphpad Prism 9 was used for plotting and data analysis. The P-value was adjusted following Benjamini & Hochberg (BH) method. A P-value less than 0.05 was considered statistically significant if not specified above.

### Ethics Statement

The experimental protocol was approved by the Committee on the Ethics for Clinical Research Projects of Zhongnan Hospital, Wuhan, China (Approval number: 20210007).

## Results

### Data Downloaded and Pre-Processing

We downloaded three mRNA expression matrixes and one scRNA sequencing matrix associated with this study in the GEO database and partially merged them. The GSE95849 matrix was a T2DM-related dataset (29). The GSE93213 and GSE93215 matrixes consist of the gene expression data of fracture non-union and fracture healing, respectively (30). Both matrixes were generated by the same platform annotation chip, so we considered that there were no data differences between groups. GSE142786 matrix contains single-cell gene expression profiles derived from 2 samples from patients 0 days post-fracture and 30 days post-fracture (31). Details about these databases and samples can be found in **Table 1**.

**Table 1:** Clinical and demographic characteristics of the samples in the GEO database.

Database	Molecule	Subgroup	Number	Gender	Age (y)	Fasting glucose (mmol/L)	Platform	Publication
**GSE95849**	mRNA	Normal	6	Female	51.17 ± 6.08	5.10 ± 0.21	GPL22448	(27)
		T2DM	6	Female	53.83 ± 10.80	9.23 ± 2.02		
**GSE93213 & GSE93215**	mRNA	Fracture healing	19	Female (10), male (9)	40.42 ± 16.76	–	GPL6244	(28)
		Non-union	2	Female (1), male (1)	46.50	–		
**GSE142786**	scRNA	Fracture	2	–	–	–	GPL24676	(29)

After the raw data were normalized by the RMA method, we screened DEGs using the above criteria. A total of 1554 T2DM-related genes were identified, of which 1020 genes were up-regulated and 534 genes were down-regulated. Volcano plots were drawn to represent the DEGs between normal samples and T2DM patients (**Supplementary Material, Figure 1**). Heat maps were used to show the top 50 genes with the greatest differences in up-regulation and down-regulation expression, respectively (**Supplementary Material, Figure 2**). The list of all the DEGs in T2DM is shown in **Supplementary Materials Table 1**.

### Data Calculation and Determination of Trait-Related Modules in T2DM

**Figure 2:**
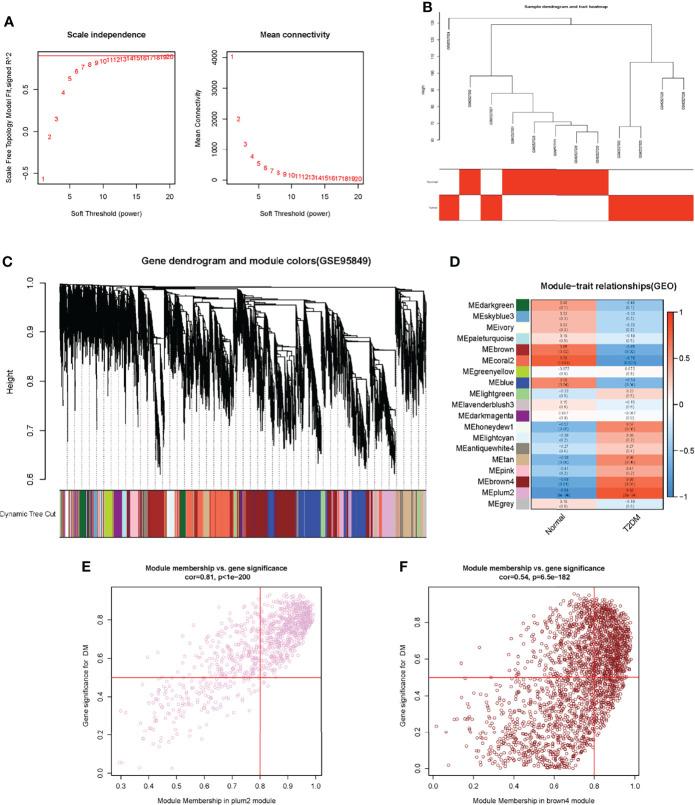
Construction of WGCNA in GSE95849. **(A)** Determination of soft threshold. **(B)** Elimination of outliers and sample filtering. **(C)** Dendrogram of all expressed genes clustered based on a dissimilarity measure (1-TOM). **(D)** Heatmap of the correlation between module eigengenes and clinical traits in DM. **(E)** and **(F)** Scatter plots of the degree and P-value of Cox regression in dataset. The x-axis indicated the degree of regression, the y- axis indicated the gene significance. Each circle represented a gene.

A total of 111,713 genes were screened for WGCNA after data preprocessing and elimination of genes with slight expression fluctuations in the T2DM matrix. To ensure the construction of a scale-free network, we applied empirical analysis to select the appropriate soft threshold, as shown in **Figure 2A**. The topological model fitting index and connectivity were stable when the soft threshold was equal to 11. All samples for the next analysis were identified and are shown in **Figure 2B**. Through the method of average linkage hierarchical clustering, a total of 18 modules were identified to be associated with clinical traits, as shown in the dendrogram in **Figure 2C**. The correlations between trait-related module genes and clinical traits are shown in **Figure 2D**. We found that the module with the highest correlation with T2DM was MEPlum2 (a coefficient of 0.83 and a p-value of about 0.0008). The genes in the MEdarkgreen module were highly correlated with normal individuals (a coefficient of 0.78 and a p-value of about 0.003). Next, we selected genes with high correlation coefficients in the module of MEPlum2 and then filtered the genes according to the cut-off criteria of |MM| ≥ 0.8 and |GS| ≥ 0.5 (32). These genes were used to identify key genes (**Figures 2E, F**). The GS and MM values of all genes are shown in **Supplementary Materials Table 2**.

### Identification of DEGs and Construction of WGCNA Network in Fracture Nonunion

**Figure 3:**
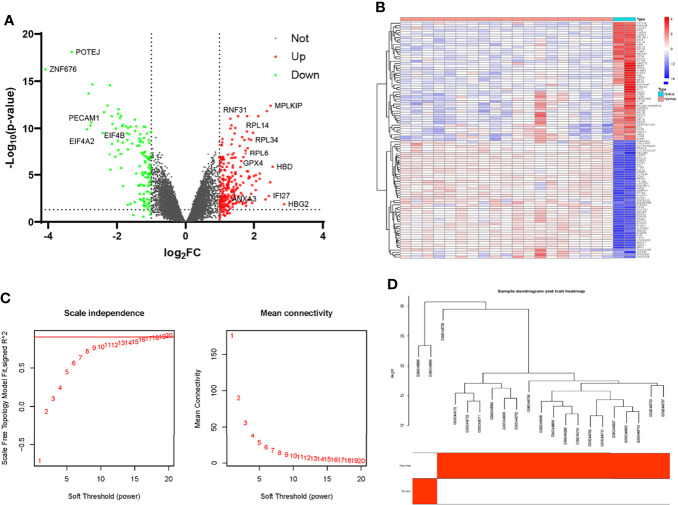
Identification of differential expressing genes and filtering of WGCNA analysis profiles in GSE93213 and GSE93215. **(A)** The volcano map of DEGs in the non-union expression matrix. **(B)** Heatmap of the top 50 over-expressed and low-expressed genes. **(C)** The selection of soft threshold during the WGCNA construction. **(D)** Filtering of outliers in the fracture non-union matrix.

**Figure 4:**
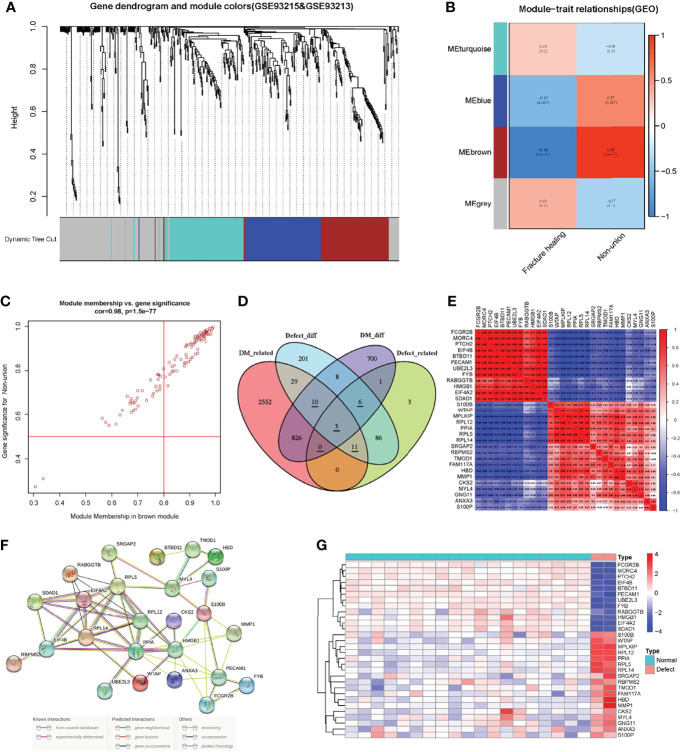
The construction of WGCNA network in the fracture non-union matrix and the identification and correlation analysis of key genes. **(A)** Dendrogram of all expressed genes clustered based on a dissimilarity measure (1-TOM) in non-union. **(B)** Heatmap of the correlation between module eigengenes and clinical traits in non-union. **(C)** Scatter plots of the degree and P-value of Cox regression in MEbrown module. **(D)** The Venn diagram showed the identification of intersection genes. **(E)** Correlation analysis of key genes in the non-union matrix. **(F)** PPI network of key genes in STRING database. **(G)** Expression heatmap of key genes in non-union matrix.

After normalization of the nonunion data, we identified DEGs according to the criteria established above. There were totally 354 DEGs, including 219 up-regulated genes and 135 down-regulated genes. As shown in **Figure 3A**, the red scatters indicate up-regulated genes, and the green scatters represent down-regulated genes. The expression of the top 50 up- and down-regulated genes in each sample is shown in **Figure 3B**. The list of all the DEGs is shown in **Supplementary Material Table 3**. Next, we applied the same method to construct the WGCNA network. After removing genes with slight expression fluctuations, a total of 524 genes were used for WGCNA analysis. When the soft threshold was equal to 16, the topological model fitting index and connectivity were good (**Figure 3C**). A total of 21 samples were used for the next analysis (**Figure 3D**). The gene clustering process is shown in **Figure 4A**. Four trait-related modules were finally identified. Surprisingly, genes in the MEbrown module correlated extremely well with fracture nonunion, of which the correlation coefficient in the module heatmap was 0.99 and the P-value was 7e-17 (**Figure 4B**). Similarly, the characteristic genes were selected according to the cut-off criteria of |MM| ≥ 0.8 and |GS| ≥ 0.5 (**Figure 4C**). The GS value and MM value of all genes are shown in **Supplementary Materials Table 4**.

### Acquisition of Intersection Genes and Correlation Analysis

To try to find the potential molecular mechanisms between T2DM and fracture nonunion, we used the intersection of the DEGs and trait-related module genes. In these four gene sets, there were 30 genes in three gene lists, of which three genes were associated with T2DM and fracture non-union and expressed differently (Related genes were underlined in **Figure 4D**). Also, 839 T2DM-related DEGs and 106 nonunion-related DEGs were identified, respectively (**Supplementary material Figure 3**, **Figure 4**). First, we extracted the expression of 30 intersection genes, and drew a correlation heatmap based on the matrix profile (**Figure 4E**). The correlations between these genes were strong. Then we searched in the STRING online database to analyze correlations of these genes (**Figure 4F**). The differences heatmap of the expression in these genes between nonunion group and normal were obvious, as shown in **Figure 4G**. Heatmaps of genes expression in the T2DM and non-diabetic groups are shown in **Supplementary Materials Figure 5**.

### GO Functional Annotation and GSEA Analysis

**Figure 5:**
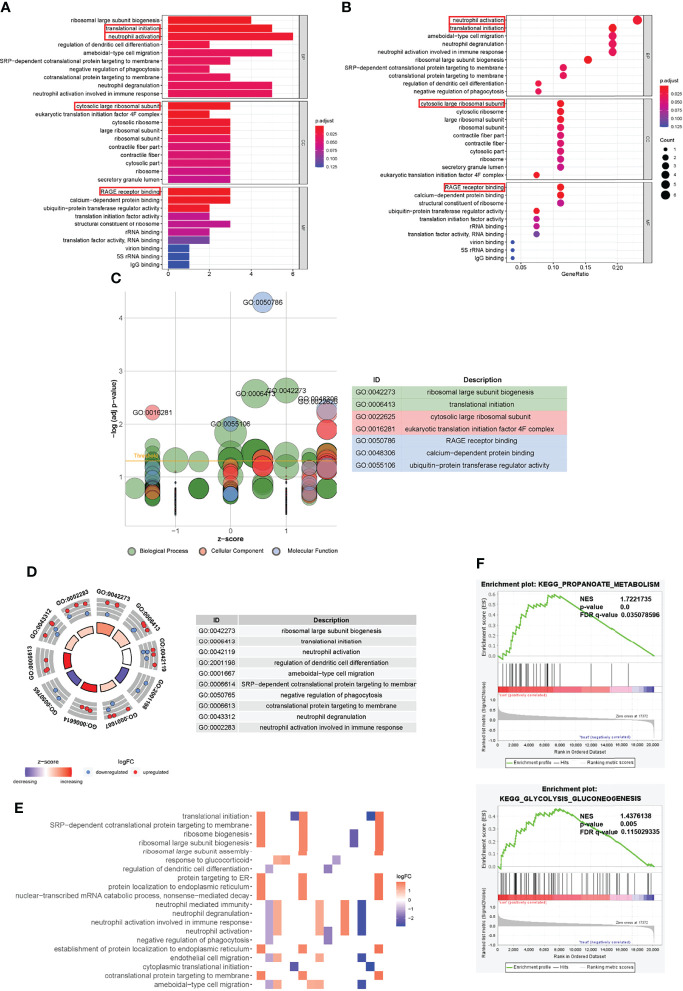
Gene Ontology functional annotation of trait-related genes and Gene set enrichment analysis. **(A, B)** GO enrichment analysis results of characteristic genes. **(C)** GOBubble plot of z-score calculation of enrichment pathways based on expression level of genes. **(D)** The outer circle presented the scatter plot of each logFC of the pathway genes. Red meant over-expression, and blue displayed decrease. **(E)** Heatmap of correlation between trait-related genes and pathways. **(F)** GSEA analysis illustrated that fracture non-union was related to propanoate metabolism and other biological metabolisms. The relevant parameters were shown.

Furthermore, we performed biological enrichment analysis of these aforementioned genes. First, we performed GO and KEGG analyses on the characteristic-related DEGs in T2DM. These genes were mainly related to neutrophil activation and neutrophil-mediated activation (biological processes, BP), early endosome and secretory granule membrane (cellular component, CC), and cytokine receptor activity (molecular function, MF) (**Supplementary Materials, Figure 6**). In the KEGG analysis, these genes were mainly enriched in the endocytosis pathway. Similarly, we performed GO and KEGG analyses of the characteristic-related DEGs in fracture non-union. It was found that these non-union genes were mainly associated with BP of viral transcription and translational initiation, CC of cytosolic part and cytosolic ribosome, and MF of the structural constituent of ribosome (**Supplementary Materials Figure 7**). In the KEGG analysis, these genes were mainly enriched in the “ribosome” pathways. Then in the same way as the hub genes, neutrophil activation had the highest correlation in BP, which suggested that the fracture non-union may be related to neutrophil-mediated immunity. In addition, the translational initiation process was also highly correlated. The CC of these genes was mainly enriched in the process of ribosomal subunit and eukaryotic translation initiation factor 4F complex. The MF was mainly related to RAGE receptor binding and calcium-dependent protein binding. The receptor for advanced glycation end products (RAGE) is a new member of the immunoglobulin superfamily, which is involved in the occurrence and development of chronic complications of diabetes. It is also associated with inflammation, tumor invasion and metastasis, and nerve regeneration (33, 34). The calcium-dependent protein binding function may be related to cellular signal transduction and various biological processes (**Figures 5A, B**) (35, 36). As shown in **Figure 5C**, a larger z-score indicated the presence of more up-regulated genes that were enriched in pathway. Similarly, we identified other immune-related pathways such as regulation of dendritic cell differentiation, negative regulation of phagocytosis, neutrophil degranulation and so on (**Figure 5D**). In addition, the heatmap shows the expression between related genes and pathways (**Figure 5E**). The complete GO enrichment results are shown in **Supplementary Materials Table 5**. Meanwhile, to understand the underlying molecular mechanisms of fracture nonunion and to explore potential links with the key genes, we conducted GSEA analysis according to the fracture healing groups and non-union groups. We found that they were associated with propanoate metabolism, s glycolysis-gluconeogenesis and some other pathways (**Figure 5F**).

### GWAS Correlation Analysis and Gene Expression Level

**Figure 6:**
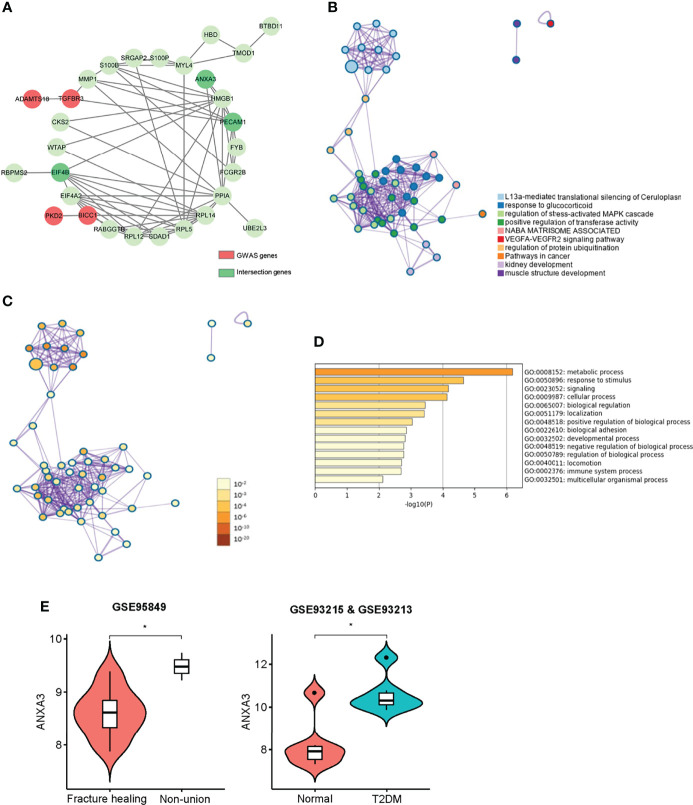
Interactions between module hub genes and genome-wide-associated genes. **(A)** The protein and protein interaction network of disease susceptibility genes and key genes in WGCNA. Red represented GWAS genes, and green represented network-related genes. **(B)** Gene enrichment clusters and nodes coloured by cluster ID. **(C) **Statistical analysis of gene enrichment clusters, the darker the colour of the node, the more statistically significant. **(D)** GO enrichment list of GWAS genes and key genes in Metascape. **(E)** The violin plot of ANXA3 gene expression level in, non-union and fracture healing patients, normal and T2DM patients. *P < 0.05.

In a previous genome-wide association study, we identified two manuscripts related to fracture healing, so we extracted these genes (*ADAMTS18*, *TGFBR3*, *PKD2*, *BICC1*) (37, 38). To explore the correlation between hub genes and susceptibility genes in the GWAS study, we performed PPI network analysis and functional correlation analysis. Our results showed that there were some biological connections between susceptibility genes and the GWAS genes (**Figure 6A**). Comparative analysis of functional enrichment was then conducted. In **Figure 6B**, gene enrichment clusters and nodes were colored by cluster ID. L13a-mediated translational silencing of ceruloplasmin expression, response to glucocorticoid and regulation of stress-activated MAPK cascade present to be more enriched based on nodes number. Correlation between nodes and enrichment pathways were displayed in **Figure 6C**. The darker the color of the node, the more statistically significant. The main processes related to susceptibility genes are displayed in **Table 2**, such as blood vessel development pathway contained the core genes (*ANXA3*, *PECAM1*) and susceptibility GWAS genes (*PKD2*, *TGFBR3*). Some cellular pathways and biological processes were also involved, such as MAPK cascade, JNK cascade, protein phosphorylation. “Metabolic process” and “response to stimulus” were mainly associated with these genes (**Figure 6D**). In conclusion, core genes may be involved in some biological processes in fracture healing, among which *ANXA3* and *PECAM1* seem to be involved in more biological processes. Next, we extracted gene expression levels of these genes in T2DM and nonunion, and found that *PECAM1* was low-expressed in nonunion and high-expressed in T2DM. The highly expressed *ANXA3* was considered to be highly expressed in both non-union and T2DM, and was used for the next analysis (**Figure 6E**). In addition, the area under curve (AUC) value of *ANXA3* in fracture non-union was 0.947, indicating that it present great prediction ability (**Supplementary Materials Figure 8**).

**Table 2:** Major enrichment analysis results associated with GWAS genes in Metascape.

GO	Description	Enrichment	Z-score	Gene list
**GO:0001568**	blood vessel development	5.813533256	4.524072956	*ANXA3|HMGB1|PECAM1|PKD2|TGFBR3*
**GO:0048514**	blood vessel morphogenesis	5.178868054	3.717047335	*ANXA3|HMGB1|PKD2|TGFBR3*
**GO:0051271**	negative regulation of cellular component movement	7.270876743	4.054934898	*HMGB1|TGFBR3|SRGAP2*
**GO:0032872**	regulation of stress-activated MAPK cascade	18.24555053	8.105323561	*FCGR2B|HMGB1|PPIA|TGFBR3*
**GO:0046328**	regulation of JNK cascade	19.91352063	7.361399195	*FCGR2B|HMGB1|TGFBR3*
**GO:0043408**	regulation of MAPK cascade	5.232179931	3.744796479	*FCGR2B|HMGB1|PPIA|TGFBR3*
**GO:0001934**	positive regulation of protein phosphorylation	6.109001293	4.681275622	*CKS2|HMGB1|PECAM1|PKD2|PPIA*
**M5885**	NABA MATRISOME ASSOCIATED	5.921908044	4.58231838	*ANXA3|MMP1|S100B|S100P|ADAMTS18*
**R-HSA-1474244**	Extracellular matrix organization	8.865155364	4.600806301	*MMP1|PECAM1|ADAMTS18*

### Single-Cell RNA Sequencing Analysis in GSE142786

**Figure 7:**
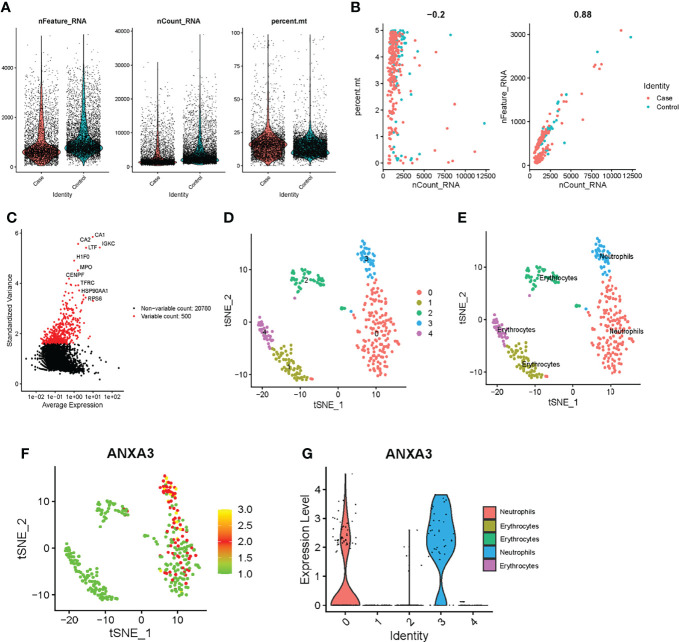
Exploring the role of *ANXA3* in fracture healing based on single-cell sequencing analysis. **(A)** The percentage of mitochondrial genes and low gene expression cells filtering. **(B)** Evaluating the correlation of sample genes counts with feature genes. **(C)** Calculating gene expression differences. Red dots were the most significant top 500 genes. **(D)** Five cell clusters were identified. **(E)** Cell clusters were mainly annotated as neutrophils and erythrocytes. **(F)** Scatter plot of *ANXA3* expression in these cluster cells. **(G)** *ANXA3* was high expressed in neutrophils in violin plot.

**Figure 8:**
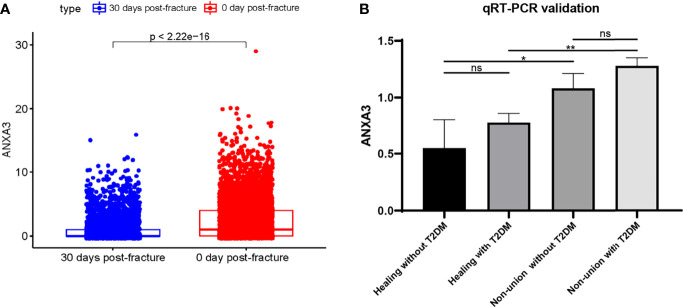
Validation of *ANXA3* expression level. **(A)** The *ANXA3* expression level was lower with increasing days post-fracture physiologically **(B)** *ANXA3* illustrated higher expression levels in patients with T2DM and non-union by qRT-PCR (n=3, each group). ns, no significance; *P < 0.05, **P < 0.01.

We performed data filtering and evaluation on single-cell dataset. The samples in the single-cell data were obtained from bone and bone marrow tissue in the fracture site of patients 0-day and 30-day post femoral fracture which represent the physiological fracture healing process. We aimed to figure out the cell types and gene expression level alternations at the fracture healing site using this data set. Then, we further explored the role and expression level of the biomarker gene *ANXA3*. As shown in **Figure 7A**, we firstly removed cells with low expression levels by above filtering criteria. The correlation between the count of sample genes and feature genes was 0.88, indicating that sequencing depth was reasonable (**Figure 7B**). We next selected the 500 genes that were highly variable in these cells and top 20 genes were labelled in **Figure 7C**. And cells can be enriched into 5 clusters in **Figure 7D**. They were mainly annotated as neutrophils and erythrocytes (**Figure 7E**). The marker gene *ANXA3* was mainly expressed in neutrophil-annotated clusters, and was less expressed in other cluster cells (**Figures 7F, G**), suggesting that *ANXA3* was mainly associated with neutrophils during fracture healing. Further, we found that the gene expression of *ANXA3* was significantly lower in 30 days post-fracture than that in 0-day post-fracture (p < 2.22e-16) (**Figure 8A**). The results showed that *ANXA3* associated with neutrophils was gradually down-expressed with the development of physiological fracture healing. In addition, studies have shown that *ANXA3* promotes immune processes in early stage of inflammation which facilities tissue repair, but it plays a negative role *via* regulating neutrophils when it is continually and excessively expressed (39). In our study, we found that neutrophils activation was of importance in the development of T2DM and fracture non-union (**Figures 5A, B**), and *ANXA3* was identified as a hub gene among them. More importantly, *ANXA3* was associated with neutrophils during fracture healing based on single cell analysis (**Figures 7F, G**). Based on these results, we proposed that up-regulated levels of *ANXA3* in T2DM negatively influenced fracture healing potentially by mediating neutrophils activation. *ANXA3* can be a biomarker.

### qRT-PCR Validation of Gene Expression Level

To determine the expression level of *ANXA3* in patients with T2DM and non-union, we collected blood from patients in Zhongnan Hospital and verified *ANXA3* expression levels by using RT-qPCR. The relevant clinical characteristics of each group were presented in **Supplementary Materials Table 6**, including gender, age, time after surgery and their fasting glucose results. Blood sample were fasting drawn before surgery. The qRT-PCR results showed *ANXA3* was highly expressed in patients with fracture non-union (Non-union without T2DM: P = 0.0325; Non-union without T2DM: P = 0.0013) (**Figure 8B**). And *ANXA3* gene expression may be higher in T2DM than in non-diabetic patients. Our results illustrated that *ANXA3* expression was increased in patients with T2DM and fracture non-union using high-throughput sequencing analyses and clinical sample validation. Up-regulated *ANXA3* may be indicative of non-union in T2DM patients.

## Discussion

Fracture healing delay and nonunion are difficult problems encountered in clinical practice. Despite the remarkable ability of bones to heal and regenerate without scarring, approximately 10% of fractures have healing barriers, which are common in diabetic patients (40) and smokers (41). Among them, the relationship between diabetes and healing has been of interest. On the one hand, diabetes can increase the risk of fracture by altering bone mineral density and bone microenvironment (42–44). On the other hand, it can also increase the formation of advanced glycation end products (AGEs) (45), reactive oxygen species (ROS) (46), inflammation (47), and other ways that affect osteoblasts and osteoclasts. These side effects can in turn affect fracture healing and cause other complications (40, 48). In addition, there are many *in vitro* studies showing that hyperglycemia can affect bone metabolism and bone maturation by regulating gene expression (49, 50). Some large clinical trials have shown that the progress of diabetic complications continues even when patients’ blood glucose level is well controlled (51–53). The research of genetic therapy targets provides opportunities for the development of new drugs and the patient prognosis after glucose homeostasis (54). However, to our knowledge, genomic studies of diabetes and fracture healing remain lacking. Our research aims to initially explore the potential correlation and molecular mechanisms between T2DM and non-union, and to find the core genes and biological processes that will provide some background for future studies.

In this study, we downloaded public data from GEO data to analyze the transcriptomic data, and collected patient samples from the local hospital for validation. First, we normalized the downloaded data and screened out the differential expression genes. Second, we applied the WGCNA method to identify the characteristic genes of the network module, and took intersection with the differentially expressed genes. Third, we performed GO and KEGG enrichment analyses on these genes. Fourth, we performed correlation analysis and biological function analysis of the hub genes and found that these genes were mainly associated with neutrophil activation, transcription initiation, ribosomal unit and RAGE receptor binding. Fifth, we analyzed GWAS-related genes. The main biological processes associated with GWAS genes and hub genes were metabolic process, blood vessel development, regulation of stress-activated MAPK cascade, and regulation of JNK cascade. Sixth, we employed single-cell sequencing analysis to analyze the function of biomarker genes in fracture healing, speculating that *ANXA3* may adversely affect fracture healing by regulating abnormal neutrophil activation. Finally, we verified the expression of *ANXA3* in patients with T2DM and fracture non-union by qRT-PCR.

In the course of analyzing of biological functions of key genes, some enrichment pathways, such as neutrophil activation and RAGE receptor binding related, were attracted to our attention. Many studies have demonstrated that diabetes can increase the levels of human pro-inflammatory factors, such as TNF-α, IL-1β, and IL-6. These cytokines can activate neutrophils (55, 56). AGEs are widely known in the development of diabetes and complications and are usually present in extracellular matrix to disrupt matrix-matrix and matrix-cell interactions (57). Although blood glucose levels are well controlled, AGEs can remain high in the tissues of diabetic patients for a long time (58), directly affecting the quality and fracture healing, and increasing the level of RAGE expression (59, 60). It has been shown that the combination of AGEs and RAGE will activate NAPDH oxidase and promote intracellular ROS formation (61), which, in return, will enhance AGE-mediated activation of the transcription factor nuclear factor-kappa B. These reactions will further aggravate inflammation and inhibit bone accumulation (60, 62). In the GSEA enrichment analysis, the nonunion group is mainly enriched in phosphate metabolism. As we know, there are many cellular signaling pathways related to it, such as the well-known PKC signaling pathway which is highly related to T2DM damage (63, 64). Furthermore, when analyzing the biological correlation between key genes and GWAS genes, we found that blood vessel development, blood vessel morphogenesis and extracellular matrix organization pathways may be related to them. The damage to blood vessels in diabetic patients will affect the development of osteoblasts in the hematopoietic niche and the delivery of osteoblasts and osteoclasts to the bone remodeling unit (65, 66). However, studies on *ANXA3* have focused more on tumors. In our study, *ANXA3* was identified as a trait-related gene associated with T2DM and nonunion, and were highly expressed. And we found that *ANXA3* was mainly associated with neutrophils, and its expression decreased in 30 days post-fracture. It has been reported that *ANXA3* can activate neutrophils, promote immune processes and prolong neutrophils survival (39). GO enrichment results also illustrated that neutrophil activation was closely related to T2DM and nonunion, so we speculated that up-regulated *ANXA3* may lead to fracture non-union in T2DM patients by mediating neutrophil activation. *ANXA3* is a biomarker gene and can be a therapeutic target in fracture healing and T2DM.

There are still some limitations of our study. First, high-throughput sequencing studies of diabetes patients with delayed union or fracture non-union are needed. In the process of downloading databases, we found that there was a lack of genomics studies on this theme, so we combined several related databases for analyses. However, in fact, high-throughput sequencing including bulk sequencing and single-cell sequencing data has been widely performed and provided research assistance in many tumor and non-tumor diseases research in recent years. Secondly, a larger sample size needs to be enrolled in future research. In our study, we found very limited samples, sequencing data containing more samples will provide more analyses and validation, and can also be used to construct prognostic signatures. Finally, molecular biology-based experiments are needed to confirm our findings. More experiments will help to clarify the role of *ANXA3* gene in the mechanism of T2DM and nonunion.

## Data Availability Statement

The datasets presented in this study can be found in online repositories. The names of the repository/repositories and accession number(s) can be found in the article/**Supplementary Material**.

## Ethics Statement

The studies involving human participants were reviewed and approved by The experimental protocol was approved by the Committee on the Ethics for Clinical Research Projects of Zhongnan Hospital, Wuhan, China (Approval number: 20210007). The patients/participants provided their written informed consent to participate in this study.

## Author Contributions

CL, DZ and AY contributed to conception and design. CL, YL, and YY analyzed the data. CL, YZ and YL validated the method and data. CL wrote this manuscript. DZ and AY edited the manuscript and provided constructive comments. All authors read and approved the final manuscript.

## Funding

All funding for this study was provided by Hubei Province Leading Medical Talent Project (No. LJ20200405).

## Conflict of Interest

The authors declare that the research was conducted in the absence of any commercial or financial relationships that could be construed as a potential conflict of interest.

## Publisher’s Note

All claims expressed in this article are solely those of the authors and do not necessarily represent those of their affiliated organizations, or those of the publisher, the editors and the reviewers. Any product that may be evaluated in this article, or claim that may be made by its manufacturer, is not guaranteed or endorsed by the publisher.

## Supplementary Material

Supplementary Figure 1 The volcano map of DEGs in the T2DM expression matrix.

Supplementary Figure 2 Heatmap of the top 50 over-expressed and low-expressed genes in T2DM.

Supplementary Figure 3 The Venn diagram showed the identification of intersection genes in T2DM.

Supplementary Figure 4 The Venn diagram showed the identification of intersection genes in non-union.

Supplementary Figure 5 Expression heatmap of key genes in T2DM matrix.

Supplementary Figure 6 GO and KEGG enrichment analyses in T2DM. The plots above were GO results. The plots below were KEGG results.

Supplementary Figure 7 GO and KEGG enrichment analyses in non-union. The plots above were GO results. The plots below were KEGG results.

Supplementary Figure 8 The ROC of ANXA3 in fracture non-union database.

Supplementary Table 1 The list of all the DEGs in GSE95849 matrix.

Supplementary Table 2 The GS and MM values of all genes in T2DM.

Supplementary Table 3 The list of all the DEGs in T2DM in GSE93213 and GSE93215 matrixes.

Supplementary Table 4 The GS and MM values of all genes in fracture non-union.

Supplementary Table 5 The complete GO enrichment results of key genes.

Supplementary Table 6 Clinical characteristics of the samples collected in the local hospital.
