# Supplementary material for: Identification of Up-Regulated ANXA3 Resulting in Fracture Non-Union in Patients With T2DM
Source: Front Endocrinol (Lausanne). 2022 Jun 24;13:890941. doi: 10.3389/fendo.2022.890941 (PMC9263855; doi:10.3389/fendo.2022.890941)
Supplement: Supplementary Figure 1 — The volcano map of DEGs in the T2DM expression matrix. [file DataSheet_1.zip › Supplementary Materials Figures.docx]

Supplementary Material

# Supplementary Materials Figures

1. **Supplementary Materials Figure 1:** The volcano map of DEGs in the diabetes expression matrix.
2. **Supplementary Materials Figure 2:** Heatmap of the top 50 over-expressed and low-expressed genes in diabetes
3. **Supplementary Materials Figure 3:** The Venn diagram showed the identification of intersection genes in diabetes.
4. **Supplementary Materials Figure 4:** The Venn diagram showed the identification of intersection genes in non-union.
5. **Supplementary Materials Figure 5:** Expression heatmap of key genes in diabetes matrix.
6. **Supplementary Materials Figure 6:** GO and KEGG enrichment analyses in diabetes. The plots above were GO results. The plots below were KEGG results.
7. **Supplementary Materials Figure 7:** GO and KEGG enrichment analyses in non-union. The plots above were GO results. The plots below were KEGG results.
8. **Supplementary Materials Figure 8:** The ROC of ANXA3 in fracture non-union database.

##
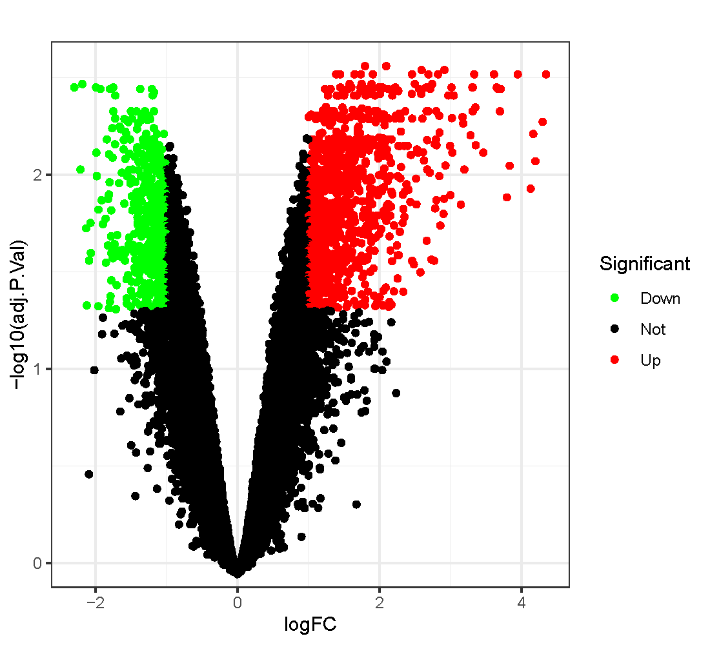


Supplementary Materials Figure 1: The volcano map of DEGs in the diabetes expression matrix.


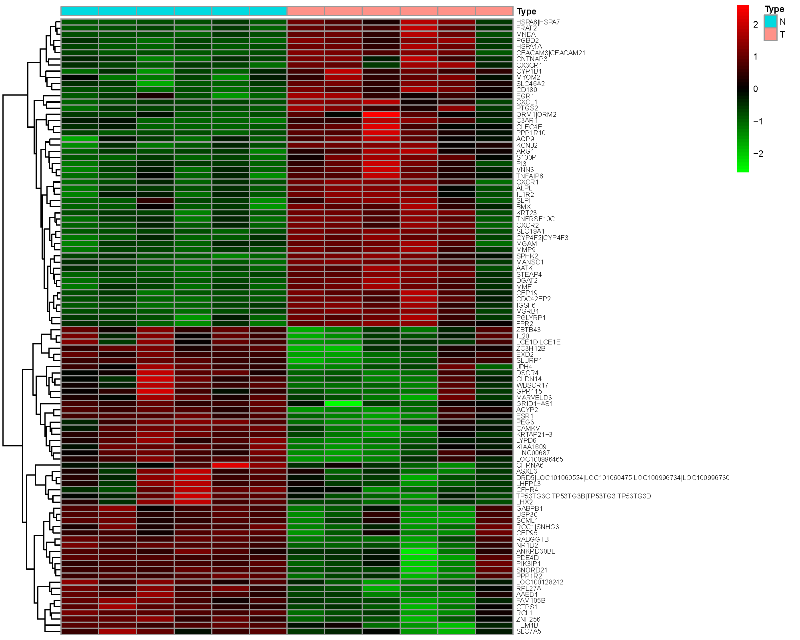


Supplementary Materials Figure 2: Heatmap of the top 20 over-expressed and low-expressed genes in diabetes


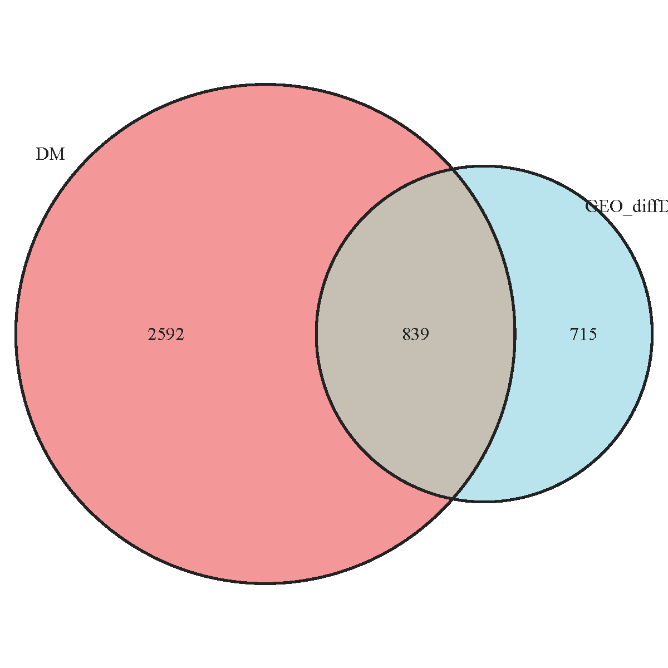

Supplementary Materials Figure 4: The Venn diagram showed the identification of intersection genes in non-union.

Supplementary Materials Figure 3: The Venn diagram showed the identification of intersection genes in diabetes.


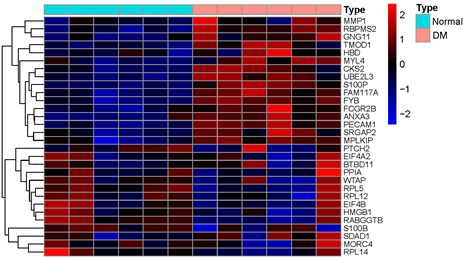


Supplementary Materials Figure 5: Expression heatmap of key genes in diabetes matrix.


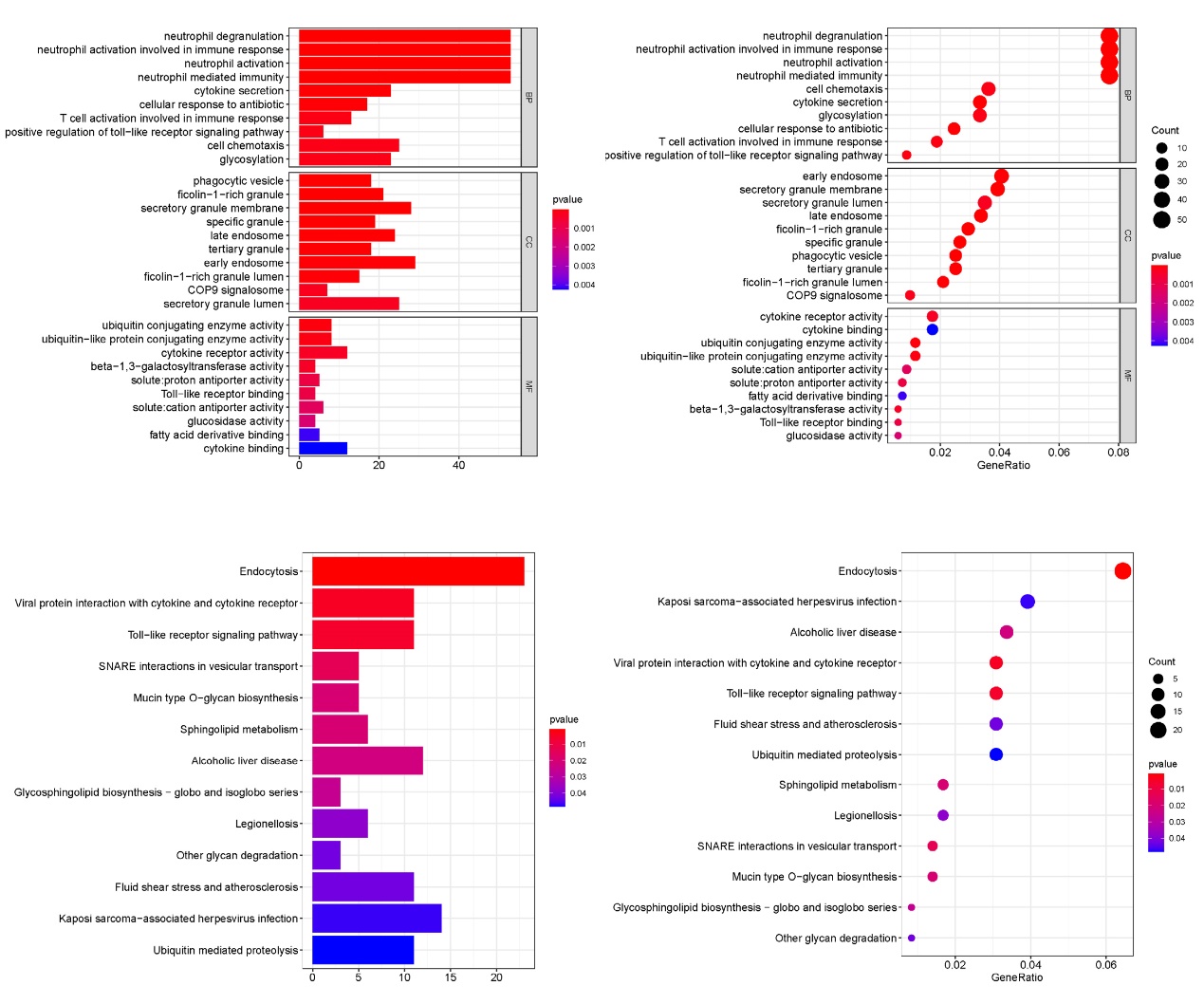


Supplementary Materials Figure 6: GO and KEGG enrichment analyses in diabetes. The plots above were GO results. The plots below were KEGG results.


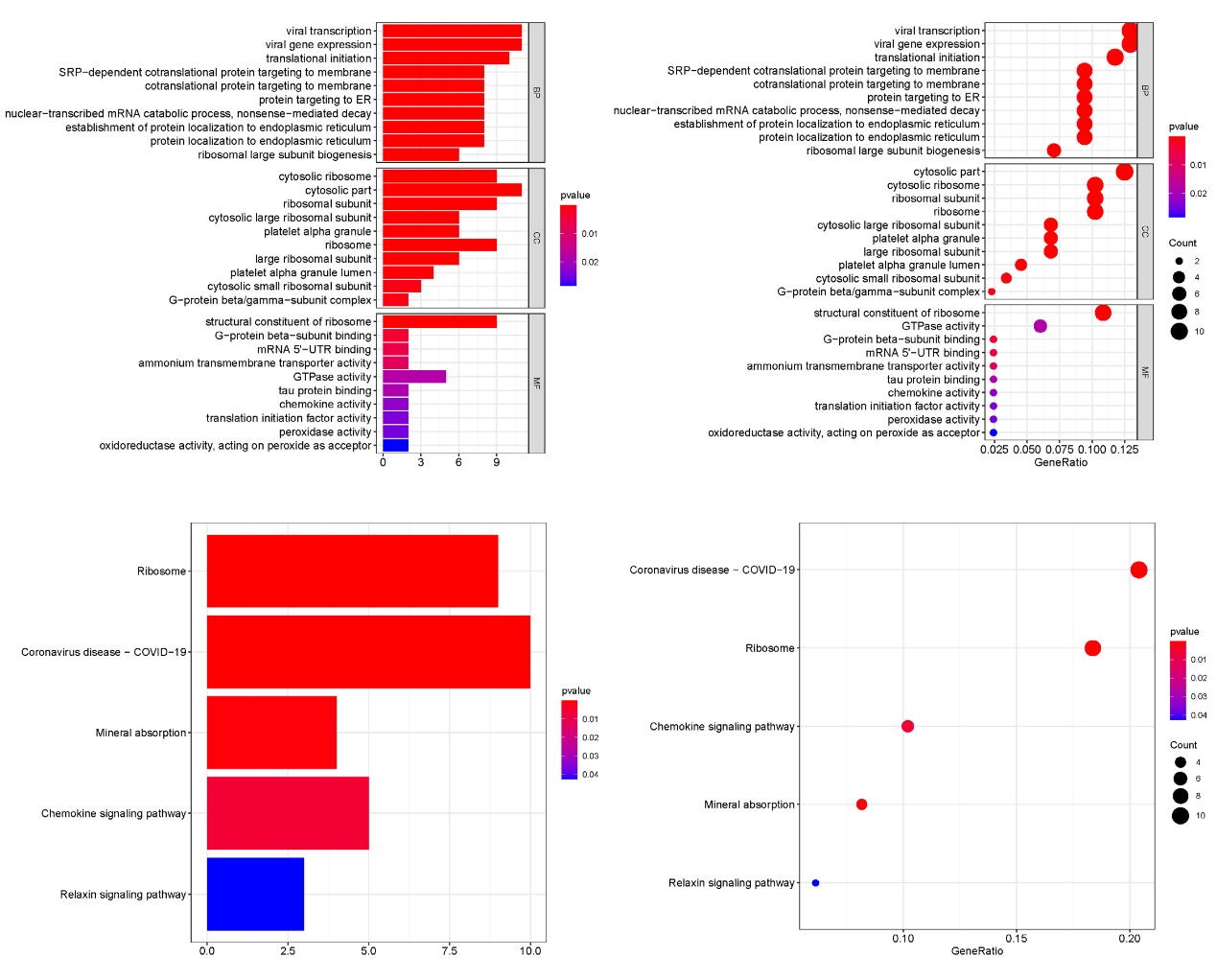


Supplementary Materials Figure 7: GO and KEGG enrichment analyses in non-union. The plots above were GO results. The plots below were KEGG results.

Supplementary Materials Figure 8: The ROC of ANXA3 in fracture non-union database.
